# Adaptations of postural sway dynamics and cortical response to unstable stance with stroboscopic vision in older adults

**DOI:** 10.3389/fphys.2022.919184

**Published:** 2022-08-29

**Authors:** Yi-Ying Tsai, Yi-Ching Chen, Chen-Guang Zhao, Ing-Shiou Hwang

**Affiliations:** ^1^ Department of Physical Therapy, College of Medicine, National Cheng Kung University, Tainan City, Taiwan; ^2^ Department of Physical Therapy, College of Medical Science and Technology, Chung Shan Medical University, Taichung City, Taiwan; ^3^ Physical Therapy Room, Chung Shan Medical University Hospital, Taichung City, Taiwan; ^4^ Institute of Allied Health Sciences, College of Medicine, National Cheng Kung University, Tainan City, Taiwan

**Keywords:** stroboscopic vision, EEG, functional connectivity, posture, visual feedback

## Abstract

**Background:** Stroboscopic vision (SV), intermittent visual blocking, has recently been incorporated into postural training in rehabilitation. This study investigated interactions of postural fluctuation dynamics and cortical processing for the elderly during stabilometer stance with SV.

**Methods:** Thirty-five healthy elderly maintained an upright stance on a stabilometer. Along with postural fluctuation dynamics, EEG relative power and EEG-EEG connectivity were used to contrast neuromechanical controls of stabilometer stance with SV and full-vision.

**Results:** Compared with the full-vision, SV led to greater postural fluctuations with lower sample entropy and mean frequency (MF). SV also reduced regional power in the mid-frontal theta cluster, which was correlated to SV-dependent changes in the size of postural fluctuations. SV also enhanced the alpha band supra-threshold connectivity in the visual dorsal and frontal–occipital loops of the right hemisphere, and the supra-threshold connectivity from Fp2 positively related to variations in the MF of postural fluctuations.

**Conclusion:** SV adds challenge to postural regulation on the stabilometer, with the increasing regularity of postural movements and fewer corrective attempts to achieve the postural goal. The elderly shift over-reliance on visual inputs for posture control with more non-visual awareness, considering deactivation of the dorsal visual stream and visual error processing.

## Introduction

Degenerative changes in the vestibular, visual, and somatosensory systems challenge postural controls of the elderly who are prone to fall risk. The age-related sensory ambiguity impacts body-sway characteristics of older adults during upright stance, resulting in a greater sway area (or energy) of the center of pressure (COP) than that of young adults (Quijoux et al., 2021). In the presence of sensory conflicts, the mean frequency (MF) change of COP in older adults is especially evident in the anteroposterior (AP) direction, corresponding to the uncertainty of balance control (Moon et al., 2021). A relatively low level of COP entropy measures is noted in older adults, which indicates functional consequences of age-related complexity loss and inferior adaptive capacity to environmental constraints (Manor et al., 2010; Walsh, 2021). An increase in COP regularity also reflects greater attentional investment in postural control for the elderly (Donker et al., 2007). The changes in COP dynamics of older adults underlie the reorganization of cortical activities to solve balance constraints due to sensory ambiguity. The elderly exhibit more widespread oscillatory activities in the alpha, beta, and gamma bands than do young adults during static balance tests (Rubega et al., 2021). Compared to young adults, the elderly show lower increases in alpha activity without significant potentiation of the high-frequency beta wave when standing with their eyes closed (Lin et al., 2021). Also, eye closure leads to stronger frontoparietal-occipital connectivity but weaker fronto-tempo-motor connectivity of the theta, alpha, and beta bands in older adults (Chen et al., 2021b). These findings highlight the fact that brain activation in older adults during bipedal stance is reorganized in response to complete visual occlusion (Chen et al., 2021b; Lin et al., 2021). In fact, older adults are less capable of redistributing the relative sensory weighting to non-visual channels for postural control, as those non-visual sensory systems are more susceptible to degenerative changes (Bugnariu and Fung, 2007; Franz et al., 2015).

Flickering between clear and opaque frames can partially occlude visual information, resulting in an intermittent vision status known as stroboscopic vision (SV). With SV, people are forced to utilize incomplete visual information during sports activities (Bennett et al., 2018). Anecdotal behavioral studies suggest that short-term adaptation effects from practicing with stroboscopic vision may help improve skilled or semi-skilled visuomotor activities such as badminton (Hulsdunker et al., 2021) or volleyball (Kroll et al., 2020). SV is claimed to foster visual memory (Appelbaum et al., 2012) and central vision attention (Appelbaum et al., 2011) along with increasing motion extrapolation, in contrast to monotony and attentional underload in the full-vision condition (Ballester et al., 2017). However, when subjects are not well-adapted, SV can undermine visuomotor performance due to the limited visual information available. With this negative impact, SV can be integrated into postural training for patients with anterior cruciate ligament (ACL) injury (Grooms et al., 2018) and chronic ankle instability (Kim et al., 2021; Lee et al., 2021; VanDeMark et al., 2021; Han et al., 2022). SV is advocated to expedite sensory reweighting for reducing visual reliance, especially for the patients with ACL and ankle instability who may excessively weigh the visual channel to maintain an upright stance in cases of impairment of the proprioceptive or somatosensory systems (Song et al., 2016; Grooms et al., 2018). Hence, SV can be analogized to improve balance control in older people; however, little attention has been paid to the extension of SV applications (Chen et al., 2021a). There are two major debates on the functional benefits of SV-based postural training. Conforming to the analogy of sensory reweighting (Isableu et al., 2003; Kim et al., 2020), visual information block adjusts sensory contributions to postural control. Within this context, visual attention is unloaded with SV. Alternatively, SV increases visual attention (Appelbaum et al., 2011; Appelbaum et al., 2012; Grooms et al., 2015), which reinforces timing anticipation and motion coherence with vision (Hulsdunker et al., 2021). It will be of value in resolving the theoretical debate with direct neural evidence of the effects of stroboscopic vision on attentional resource allocation during postural training in older adults.

In addition to regional activities in the sensorimotor and visual cortex, posture control involves in coordinated processing of cortical activities within these spatially distinct areas, especially when the effect of visual cues is manipulated (Chen et al., 2021a; 2021b, 2022). By examining COP dynamics, regional/inter-regional EEG features, and COP-EEG interaction, this study investigated neuromechanical control of upright balance with stroboscopic vision for older people, while they were standing on a stabilometer surface with visual feedback. For intermittent visual blocking, SV was hypothesized to alter postural mechanics and impair the monitoring and correction of postural errors through variation of the mid-frontal and reverent error-related network in the theta (4–7 Hz) band (Cavanagh et al., 2012; Ozdemir et al., 2016; Pezzetta et al., 2018) during stabilometer stance. Next, if the hypothesis of sensory reweighting is supported, SV may unload visual attention and reduce motion explorations due to enhanced synchronization of the visuomotor network in the alpha (8–12 Hz) and beta (13–35 Hz) bands due to an inhibition process (Pfurtscheller et al., 1996) and a sustained active state of the sensorimotor cortex with non-visual inputs (Lin et al., 2021), respectively.

## Methods

### Participants

Thirty-six older adults over 60 years old (20 females and 16 males; age: 65.8 ± 2.8 years) from a university campus participated in this study. One female participant failed to complete the experiment due to temporary dizziness during the experimental proceedings. Most participants were recreationally active with regular exercise habits. They had a corrected-to-normal vision (7 subjects: cataract surgery, 1 subject: glaucoma under control) and no known cognitive problems, history of falls, or diagnoses of neurological and musculoskeletal disorders requiring medication. Eleven subjects had known mild hypertension under regular medication. This study was approved by an authorized institutional human research review board at the University Hospital (A-ER-107-099-T). All subjects signed the consent form before the experiment, in accordance with the Declaration of Helsinki.

### Experimental procedures

**FIGURE 1:**
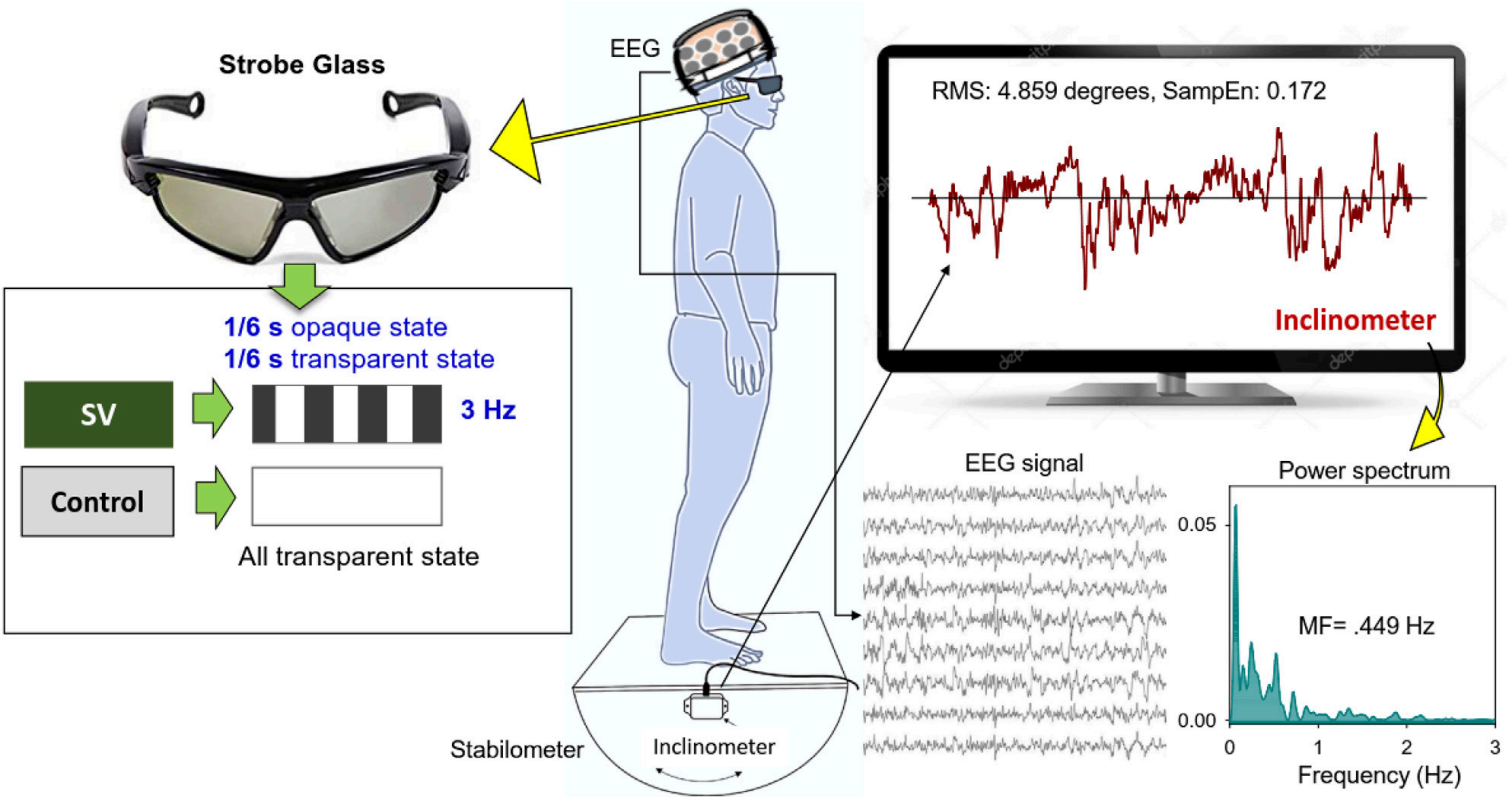
System setup and stabilometer stance with and without stroboscopic vision (SV). Under SV, the subjects regulated upright stance on the stabilometer with intermittent visual feedback of stabilometer movements at 3 Hz. In the control condition, the subjects wore stroboscopic glasses and visually tracked the stabilemeter movement trajectory with full vision.

This study used a randomized, repeated measures design. Stabilometer stance, which is commonly used to train stance stability in elderly and neurological patients with balance dysfunction, served as the postural task in this study. Protected by a custom-made wooden fence, the subjects were asked to stand barefoot on a 50 cm × 58 cm wooden stabilometer (radius: 25 cm; height: 18.5 cm) (Figure 1). The stabilometer could rotate along its sagittal axis (roll maximum excursion: 20°). Subjects needed to keep the stabilometer in a horizontal position with a real-time stabilometer trajectory and a horizontal target visual feedback on the computer monitor. The subjects wore a pair of stroboscopic glasses (Visionup Athlete VA11-AF, Japan) and attempted to couple the stabilometer movement trajectory with the horizontal target line during the stabilometer stance. In the full vision condition, the stroboscopic glasses provided no opaque state, so no visual information was occluded. In the SV condition, the stroboscopic glasses provided a 1/6-s opaque state and a 1/6-s transparent state at a rate of 3 Hz to reduce half amount of visual feedback (Figure 1). The opaque/transparent ratio was 1:1. Each trial lasted 45 s. All participants completed three experimental trials for both the SV and full vision conditions in alternating order with rest periods of 3 min between trials to prevent fatigue. Half of the participants began with an SV trial and the other half began with a full vision trial.

### Instrumentation setting

According to the 10-20 system of electrode placement, scalp EEG during stabilometer stance was recorded with Ag-AgCl electrodes and a 40-channel NuAmps amplifier (NeuroScan Inc., EI Paso, United States) with the ground electrode located along the midline ahead of F_z_. For off-line electrooculography (EOG) assessment, two electrodes were placed at the outer canthus of the left and right eyes, and two electrodes were placed infra- and supra-orbitally at the right eye. The impedances of all the electrodes were below 5 kΩ. The EEG data were recorded with a band-pass filter set at 0.1–100 Hz and a sampling rate of 1 kHz. At the same time, angular movements of the stabilometer were registered with an inclinometer (Model FAS-A, LORD MicroStrain, United States), which was fixed on the rational axis of the stabilometer. Synchronized with the scalp EEG system, the angular movements were digitized to 1 kHz with an analog-to-digital converter (Model 6341, National Instruments, United States) controlled by a Labview program (Labview v8.5, National Instruments, United States).

### Data analysis

The angular stabilometer movements were first conditioned with a 4th-order low-pass Butterworth filter (cutoff frequency: 6 Hz) and down-sampled to 100 Hz (Michalak et al., 2014). At the behavior level, a set of postural fluctuation sway metrics was derived from the stabilometer movements, including root mean squared (RMS) (degree), mean frequency (MF) (Hz), and sample entropy (SampEn). MF of postural fluctuations was derived from the power spectra of the stabilometer movement, estimated with a fast Fourier transform and the Welch method with a spectral resolution of 0.02 Hz (Figure 1). The MF reflected a spectral shift in postural sway, with higher MF indicating greater stance uncertainty. Postural sway regularity was indexed with SampEn, which is an effective biomarker to index attentional investment in postural control (Donker et al., 2007; Roerdink et al., 2011). The mathematical formula of sample entropy was $SampEn\left(m,r,N\right)=-\text{log}\left(\frac{\sum_{i=1}^{N-m}A_{i}}{\sum_{i=1}^{N-m}B_{i}}\right)$ , where *r* = 15% of the standard deviation of the force channel, *m* is the length of the template (*m* = 2), and *N* is the number of data points in the time series. *A*_*i*_ is the number of matches of the *i*th template of length *m + 1* data points, and *B*_*i*_ is the number of matches of the *i*th template of length *m* data points. The values of *m* and *r* to calculate COP complexity were identical to the settings of the previous study (Ramdani et al., 2009). Attentional involvement in postural control increases the regularity (or smaller SampEn) of stabilometer movements. The postural variables were analyzed in MATLAB R2019a software (Mathworks, United States). All of the postural variables of the three trials in the SV and control conditions were averaged for each participant.

The EEG data were first filtered between 4 and 35 Hz using a zero-phase finite impulse response (FIR) filter (60 dB/octave) to remove the DC shift and potential contaminations of low-frequency postural sway under 4 Hz. EEG data in the gamma band were not analyzed, as it was susceptible to contamination by neighboring muscle activities (van Lutterveld et al., 2017). Eye blinks in the EEG were corrected with the NeuroScan 4.3 software program (NeuroScan Inc., EI Paso, TX, United States), based on a bipolar vertical EOG channel. The EEG data were segmented into 2-s epochs after the removal of ocular artifacts. Epochs surviving automated artifact rejection were visually inspected by researchers for rejection of undetected artifacts. After that, for the regional activity of the EEG, the spectral power in each epoch was calculated using Fast Fourier Transformation (FFT) with a frequency resolution of 0.2 Hz. Relative power was computed as the ratio of the power of each spectral value to the total power. The power spectra were averaged across epochs for each channel. Based on the pooled relative power, the mean peak amplitude in each sub-band (theta (4–7 Hz), alpha (8–12 Hz), and beta (13–30 Hz)) was obtained across the EEG channels. The regions of interest for various sub-bands (theta (4–7 Hz), alpha (8–12 Hz), and beta (13–30 Hz)) were denoted on the EEG channels, where the sub-band peak amplitudes were significantly different between the SV and control conditions. Three trials of the relative powers of the electrodes in the regions of interest were averaged for the SV and control conditions for all participants. The EEG power spectrum were analyzed in MATLAB R2019a software (Mathworks, United States). In addition to regional activity, we estimated the inter-regional connectivity of the EEG of the 30 electrode pairs from all sub-bands in terms of the phase-lag index (PLI). The PLI quantifies the distribution asymmetry of phase differences in the instantaneous phases of two time-series based on the Hilbert transformation. The PLI is defined thus: PLI=|E{sgn(Δφ(t))}| , where φ(t) is the phase difference and *sgn* is a function that extracts the sign of a real number. PLI was used because it is relatively immune to volume conduction and common source noises (Stam et al., 2007). A square 30 × 30 PLI adjacent matrix for the SV and control conditions was obtained after the computation of the PLI across all electrode pairs**.** The PLI-based functional connectivity was calculated with the HERMES function in Matlab (Niso et al., 2013).

### Statistical analysis

EEG variables and the postural variables in the three experimental trials in the SV and control conditions were averaged for each subject. Hotelling’s T-squared statistics were used to examine the intermittent visual effect (SV vs. control) on the postural fluctuation characteristics (RMS, MF, and SampEn). The post-hoc test for the postural variables was a paired t-test using Bonferroni correction. For EEG variables of regional activity, paired t-test was used to contrast differences in the pooled relative powers of each sub-band for the EEG channels in the regions of interest between the SV and control conditions. The strength of each connection within the PLI adjacent matrix in the SV and control conditions was first examined with a two-sample t-test. A set of supra-threshold edges ( $\left|t_{34}\right|>2.728$ , *p* < 0.005) was extracted to highlight the differences in topological distributions between the SV and control conditions. This identified all possible connected components, or subnetworks, in the matrix at this uncorrected level. In the framework of network statistics, a permutation test was performed 5,000 times to examine variations in the null distribution of the supra-threshold connectivity between the SV and control conditions. Methodological details of corrected network-based statistics were documented in the work of Zalesky et al. (2010). In fact, SV enhanced or suppressed the supra-threshold connectivity. For various EEG sub-bands [theta (4–7 Hz), alpha (8–12 Hz), and beta (13–30 Hz)], we further calculated the mean PLI for the supra-threshold connectivity of the electrode pairs that exhibited greater connectivity strengths (SV > control) and smaller connectivity strengths (SV < control). Pearson’s correlation was performed to examine the significance of correlations between the normalized differences (ND) in postural sway metrics between the SV and control conditions [(SV-control)/control)] and the ND in variations in sub-band regional activities/inter-regional connectivity. The level of significance was 0.05. Data are presented as group means ± standard deviation. All statistical analyses were performed in IBM SPSS Statistics (v.19).

## Results

Table 1 shows the results of Hotelling’s T-squared statistics comparing the postural fluctuation variables between the SV and control conditions during stabilometer stance. The postural fluctuation variables differed significantly with and without intermittent vision (Wilks’ Λ = 0.287, *p* < 0.001). Post-hoc analysis revealed that intermittent vision led to greater RMS (*p* < 0.001), smaller SampEn (*p* < 0.001), and MF (*p* < 0.001) of postural fluctuations than full vision did.

**TABLE 1:** The contrast angular fluctuation of stabilometer movement dynamics between the stroboscopic vision and control conditions.

Stabilometer movement dynamics	SV	Control	*Hotelling’s Statistics*	*Post-hoc test*
RMS (degree)	4.848 ± 2.013^†††^	2.487 ± 0.969	Wilks’ Λ = 0.287, *p* < 0.001	*t*_*34*_ = 9.036, *p* < 0.001
SampEn	0.186 ± 0.074^***^	0.225 ± 0.083		*t*_*34*_ = −3.942, *p* < 0.001
MF (Hz)	0.308 ± 0.089^***^	0.398 ± 0.139		*t*_*34*_ = −4.329, *p* < 0.001

^†††^SV > Control, *p* < 0.001.

***SV < Control, *p* < 0.001.

RMS, root mean square of angular fluctuations; SampEn, sample entropy of angular fluctuations; MF, mean frequency of angular fluctuations.

The contrast of pooled topological distributions of the relative powers of the theta (4–7 Hz), alpha (8–12 Hz), and beta (13–35 Hz) waves between the SV and control conditions during stabilometer stance is shown in Figure 2A. The theta power was the most evident in the mid-frontal areas. A pronounced alpha wave was noted in the posterior part of the cortex (especially the parietal–occipital area). Stronger beta power was noted in the prefrontal and bilateral temporal areas. There was visible modulation of the spatial distribution of the EEG relative power with intermittent vision. Figure 2B summarizes the results of paired t tests to contrast the relative EEG powers of the theta, alpha, and beta bands at each electrode between the SV and control conditions. The theta power was smaller in the F_z_ (*t*_*34*_ = 2.938, *p* =0.006) and left sensorimotor areas (FC_3_, C_3_, and CP_3_) (*p* < 0.05) for the SV condition (Figure 2B, top). The average relative power of three electrodes in the left sensorimotor area in the SV condition was smaller than that in the control condition (*t*_*34*_ = 2.494, *p* =0.018). The relative alpha power in the fronto-centro-parietal (FCP) area (FC_z_, C_z_, CP_z_, and P_z_) was enhanced with intermittent vision (Figure 2B middle). The mean relative alpha power of the electrodes in the regions of interest for the SV condition was greater than that for the control condition (*t*_*34*_ = -3.781, *p* =0.001). The relative beta power in the fronto-centro-parietal (FCP) area (F_z_, FC_z_, C_z_, CP_z_, and P_z_) and occipital area (O_1_, O_2_, and O_z_) was also mediated by intermittent vision (Figure 2B, bottom). The mean relative powers of the electrodes of the FCP area (*t*_*34*_ = −3.559, *p* =0.001) and occipital area in the SV condition (*t*_*34*_ = −3.347, *p* =0.002) were greater than those in the control condition.

**FIGURE 2A:**
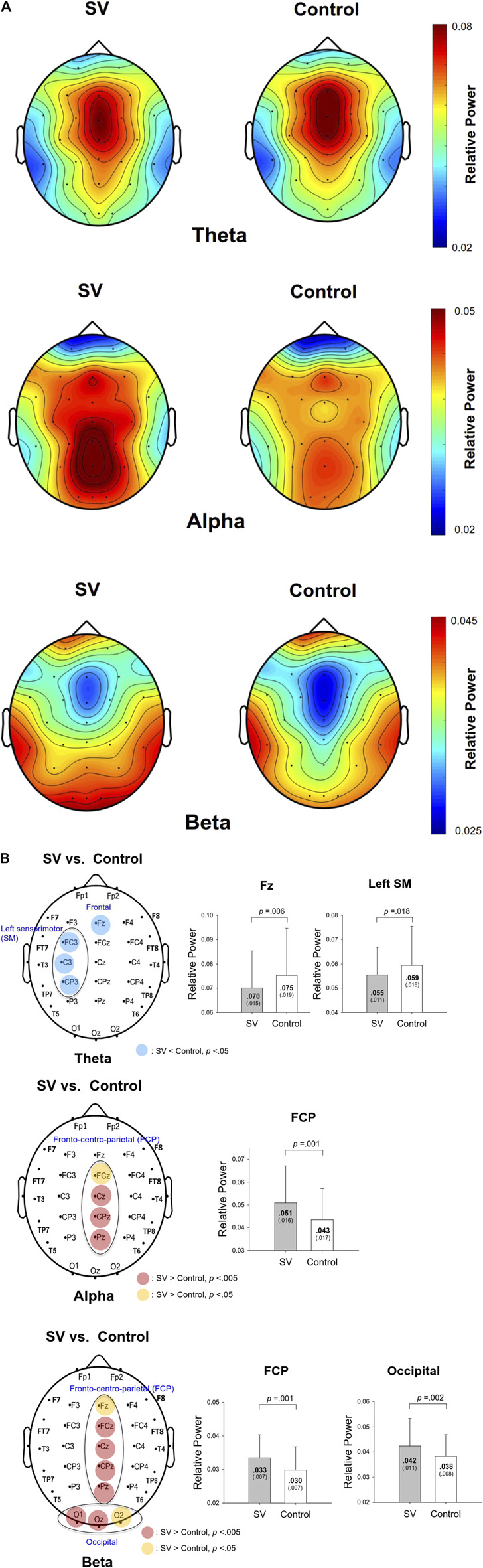
The pooled topological spectral mapping of scalp EEG at the theta (4–7 Hz), alpha (8–12 Hz), and beta bands (13–35 Hz) in the stroboscopic vision (SV) and control conditions. Oscillatory waves in the theta and alpha bands are most evident in the mid-frontal and parietal-occipital areas, respectively. Beta oscillation is most evident in the mid-frontal and bilateral temporal areas in the control condition. Relative powers of theta, alpha, and beta bands are selectively tuned to stroboscopic vision.

**FIGURE 2B:**
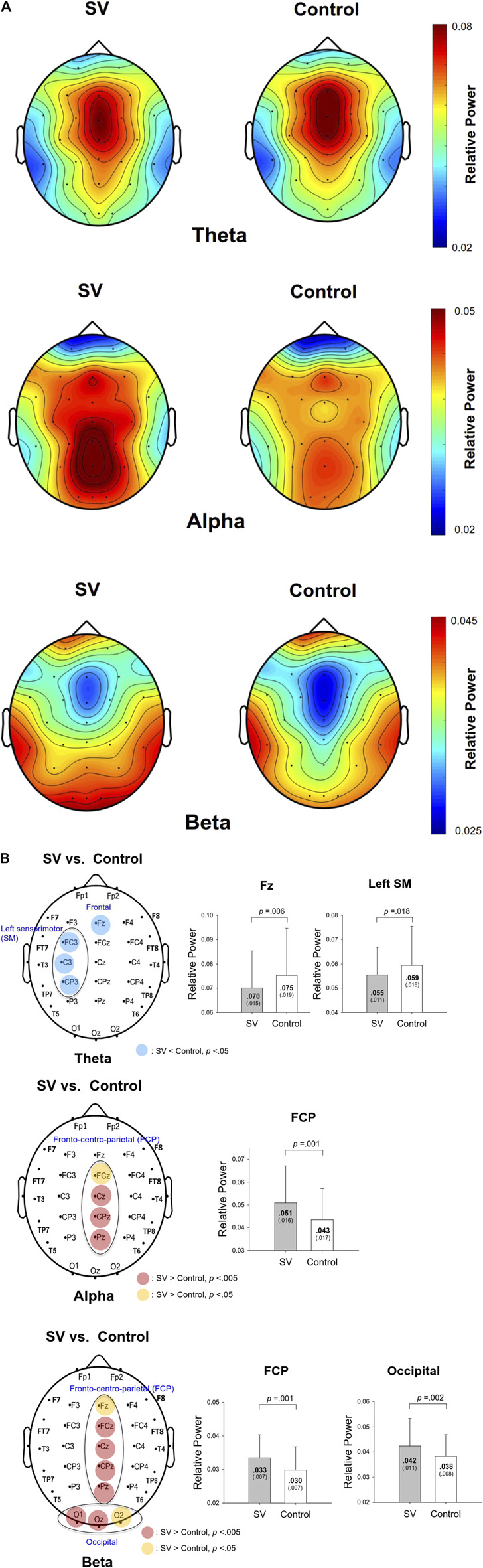
Schematic illustration of the SV-induced changes in the relative power of scalp electrodes for the theta (top), alpha (middle), and beta (bottom) bands. The right subplots of **(B)** contrast the mean relative powers at various spectral bands in the regions of interest between the SV and control conditions. A warm color indicates greater regional activity in the SV condition than in the control condition. A cold color indicates smaller regional activity in the SV condition than in the control condition.

**FIGURE 3:**
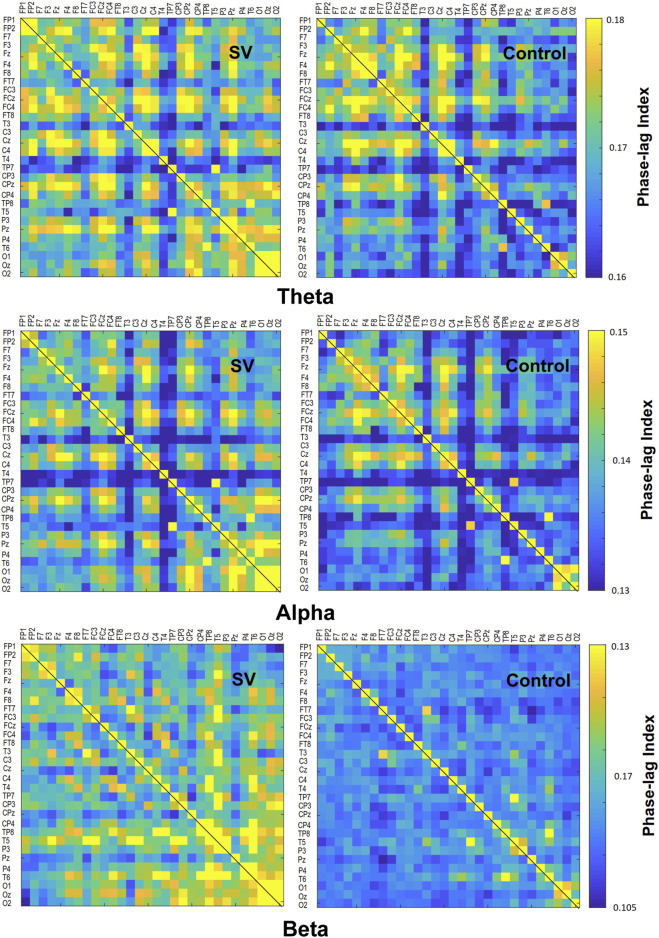
The adjacent matrices of t values contrasting the phase-lag index (PLI) values of all electrode pairs in the theta (4–7 Hz), alpha (8–12 Hz), and beta (13–35 Hz) bands between the SV and control conditions. A contrasting wiring diagram on the scalp shows the topological distributions of the supra-threshold connectivity tuned to stroboscopic vision (SV vs. Control) (|*t* | > 2.898, *p* < 0.005). (Red line: SV > Control, *p* < 0.005; Blue line: SV < Control, *p* < 0.005)

**FIGURE 4:**
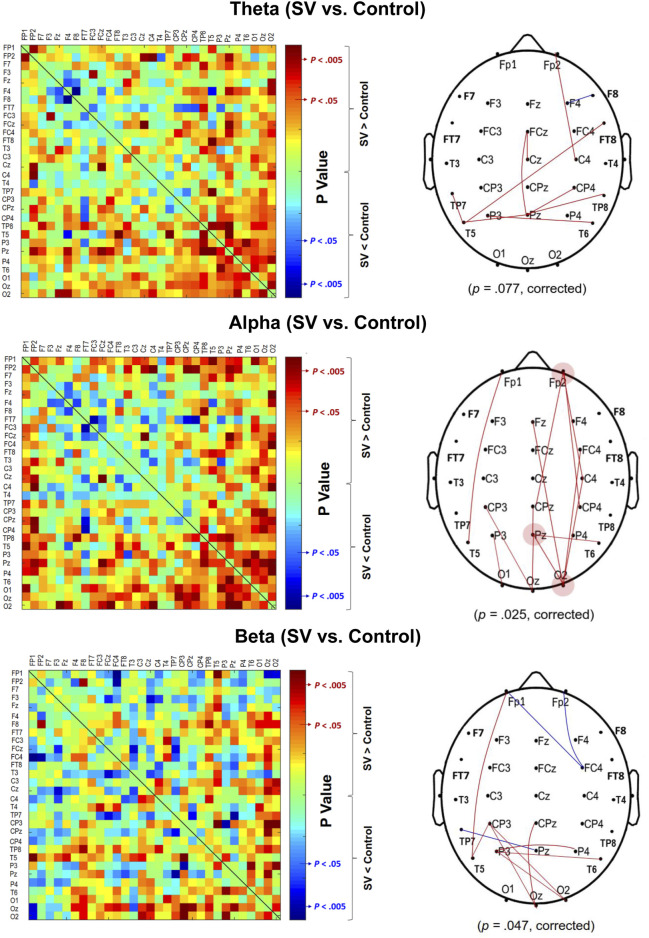
The phase-lag index (PLI) adjacent matrices represent the averaged strengths of inter-regional connectivity of the theta (4–7 Hz), alpha (8–12 Hz), and beta (13–35 Hz) bands between the SV and control conditions.

The contrast of the pooled PLI of EEG–EEG electrode pairs of the theta (4–7 Hz), alpha (8–12 Hz), and beta (13–35 Hz) waves between the SV and control conditions during stabilometer stance is shown in Figure 3. The inter-regional functional connectivity during stabilometer stance is represented with PLI values. The adjacent matrix of t-values in the left plots of Figure 4 describes the differences in PLI for all electrode pairs between the SV and control conditions at various sub-bands. According to the significance of the t-values, the supra-threshold connectivity ( $\left|t_{34}\right|>2.728$ , *p* < 0.005) of the theta, alpha, and beta bands was labeled on the scalp map (Figure 4, right plots). The supra-threshold connectivity highlighted the most evident changes in connectivity strength due to intermittent vision in older adults. For the theta band, SV mainly potentiated a few connections in the centro-parietal-temporal area and suppressed functional connectivity between F_4_ and F_8_ (Figure 4, upper right). For the alpha band, all supra-threshold connectivity was enhanced with SV (Figure 4, middle right). The results of corrected network-based statistics indicated that SV could significantly affect the EEG–EEG network of the alpha (*p =* 0.025, corrected) and beta (*p* = 0.047, corrected) bands during stabilometer stance. The EEG–EEG network in the theta band was marginally tuned to SV (*p* = 0.077, corrected). SV potentiated supra-threshold connectivity of the theta band, except for that of the F_4_–F_8_ pair, which was suppressed with SV.

**FIGURE 5:**
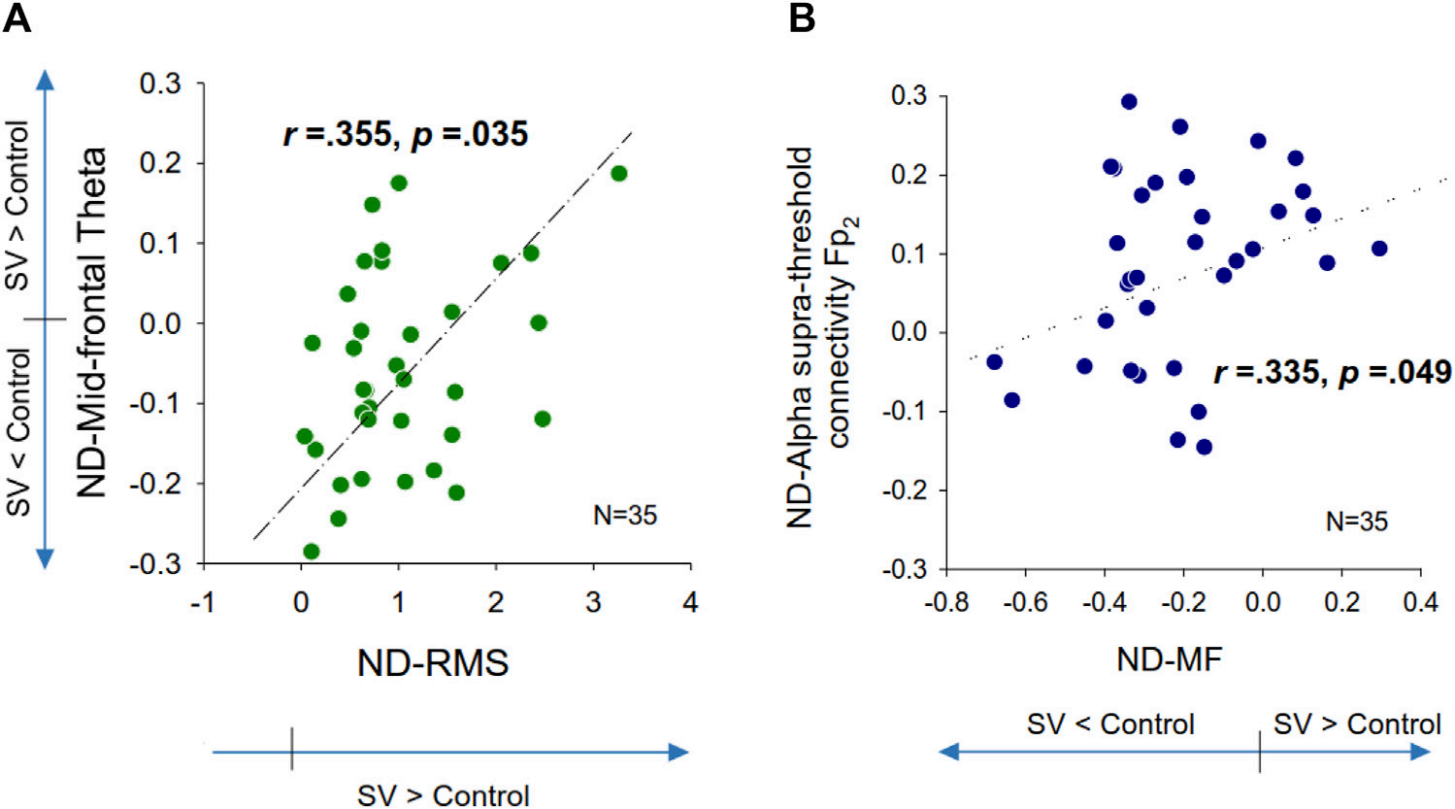
Scatter plots of normalized differences (ND) in EEG variables and normalized differences in postural fluctuation variables that are significantly correlated. **(A)** ND in mid-frontal theta vs. ND in root mean square of postural fluctuations, **(B)** ND in supra-threshold connectivity of Fp_2_ in the alpha band vs. ND in mean frequency (MF) of postural fluctuations.

Table 2 summarizes the Pearson’s correlations between normalized changes in regional activity and postural sway dynamics due to SV. Notably, there was a significant correlation between SV-dependent differences in the mid-frontal theta rhythm (ND-Theta, Frontal) and ND-RMS of postural fluctuations (*r* = 0.355, *p* = 0.035) (Figure 5A). Besides that, changes in EEG regional activity were not significantly correlated to other postural sway dynamics (*p* > 0.05). Table 3 summarizes the Pearson’s correlations between normalized increases/decreases in supra-threshold EEG–EEG connectivity of various bands and postural sway dynamics due to SV. On account of a marginally significant correlation between ND enhancement of supra-threshold connectivity of the alpha band and the ND-MF of postural fluctuations (*r* = 0.307, *p* = 0.073), a further detailed correlation analysis was performed with consideration of the ND enhancement of the supra-threshold connectivity of the alpha band from the three key nodes (O_2_, P_z_, and Fp_2_) (Table 4; Figure 5B). In addition, ND-MF of postural fluctuations was positively correlated with supra-threshold connectivity from Fp_2_ in the alpha band (*r* = 0.335, *p* = 0.049) (Table 4; Figure 5B).

**TABLE 2:** Pearson’s correlation between changes in regional spectral activity and stabilometer dynamics due to stroboscopic vision. The black-bolded fonts in the shaded areas indicate significant correlations of regional activity and postural sway dynamics.

*Pearson’s*	ND-theta		ND-alpha	ND-beta	
*Correlation* (*n* = 35)	Mid-frontal	Left sensorimotor	Fronto-centro-parietal	Fronto-centro-parietal	Occipital
**ND-RMS**	***r* = 0.355, *p* =0.035**	*r* = 0.171, *p* =0.326	*r* = −0.147, *p* =0.398	*r* = −0.263, *p* =0.126	*r* = 0.065, *p* =0.711
**ND-SampEn**	*r* = −0.135, *p* =0.349	*r* = −0.050, *p* =0.744	*r* = 0.025, *p* =0.885	*r* = −0.018, *p* = 0.918	*r* = 0.105, *p* =0.548
**ND-MF**	*r* = −0.194, *p* =0.265	*r* = −0.096, *p* =0.583	*r* = −0.096, *p* =0.596	*r* = 0.033, *p* =0.583	*r* = 0.006, *p* =0.972

ND, normalized differences; RMS, root mean square of angular fluctuations; MF, mean frequency of angular fluctuations; SampEn, sample entropy of angular fluctuations.

**TABLE 3:** Pearson’s correlation between changes in supra-threshold inter-regional coupling of spectral bands/key-nodes and stabilometer dynamics. Pearson’s correlation between changes in supra-threshold inter-regional functional coupling (*p* < 0.005) of various spectral bands and postural sway dynamics due to stroboscopic vision. The numbers within parentheses are the edge number of each key node. The black-bolded and grey-bolded fonts in the shaded areas indicate significant and marginally-significant correlations of regional activity and postural sway dynamics, respectively.

*Pearson’s*	ND-supra-threshold edge (theta)		ND-supra-threshold edge (alpha)	ND- ND-supra-threshold edge (beta)	
*Correlation* (*n* = 35)	Enhancement	Suppression	Enhancement	Enhancement	Suppression
**ND-RMS**	*r* = −0.041, *p* = 0.813	*r* = 0.276, *p* = 0.109	*r* = −0.220, *p* = 0.203	*r* = −0.089, *p* = 0.610	*r* = 0.234, *p* =0.177
**ND-SampEn**	*r* = 0.069, *p* = 0.693	*r* = −0.180, *p* = 0.299	*r* = 0.207, *p* = 0.232	*r* = 0.054, *p* = 0.756	*r* = 0.053, *p* =0.763
**ND-MF**	*r* = −0.059, *p* = 0.737	*r* = −0.216, *p* = 0.213	**r = 0.307, *p* = 0.073**	*r* = 0.236, *p* = 0.172	*r* = −0.072, *p* =0.680

ND, normalized differences.

**TABLE 4:** Pearson’s correlation between changes in supra-threshold inter-regional coupling of spectral bands/key-nodes and stabilometer dynamics. Pearson’s correlation between increases in supra-threshold inter-regional functional coupling of key nodes (O2, Pz, and Fp2) of alpha-band and postural sway dynamics due to stroboscopic vision. The numbers within parentheses are the edge number of each key node. The black-bolded and grey-bolded fonts in the shaded areas indicate significant and marginally-significant correlations of regional activity and postural sway dynamics, respectively.

*Pearson’s*	ND-supra-threshold edge (alpha)		
*Correlation* (*n* = 35)	O2 (5)	Pz (4)	Fp2 (3)
**ND-MF**	*r* = 0.154, *p* = 0.378	*r* = 0.173, *p* = 0.320	***r* = 0.335, *p* = 0.049**

ND, normalized differences.

## Discussion

Stroboscopic visual disruption led to changes in neuromechanical control of the elderly on the stabilometer stance, in light of variations in postural fluctuation dynamics, EEG regional activity, and EEG–EEG connectivity. Relative to full-vision, SV impaired postural stability, with smaller MF and SampEn of postural fluctuations in stabilometer stance. Notably, a decline in the mid-frontal (F_z_) theta oscillation and enhancement of the supra-threshold connectivity of Fp_2_ in the alpha band were correlated to SV-related changes in postural fluctuation dynamics. This study is the first to demonstrate evidence of potential postural training to reweigh sensory channels with stroboscopic vision for older adults on an uneven surface.

### Stroboscopic vision-dependent variations in postural strategy

Compared to quiet stance, stabilometer stance with additional mechanical constraints varies the available sensory information and amplifies the motor command to master balance skills (Rougier et al., 2012). Stabilometer stance is commonly used to train posture control in rehabilitation clinics. Stroboscopic vision is hypothesized to be a sensory reweighting approach by reducing visual feedback (Isableu et al., 2003; Kim et al., 2020). In agreement with previous studies (Kim et al., 2020; Wohl et al., 2021; Han et al., 2022), the increment of postural fluctuation size (Table 1) suggested that the intermittent vision feedback added to balance difficulty, because of the reduction in the availability of visual input for the older adults who favor the utilization of visual information to ensure the visuospatial demands of a posture task (Woollacott et al., 1986; McChesney and Woollacott, 2000). In addition, the SV varied postural strategy, in support of declines in mean frequency and sample entropy of postural fluctuations (Table 1). The hypothesis of intermittent motor control (Miall et al., 1993) posited that postural fluctuations stem from superimposition of centrally-scaled pulse elements to tune postural responses that deviate from the target goal. Central to this interpretation is that the older adults with SV reduced the number of corrective attempts (or reduced the frequency of motor explorations) for postural corrections (Paillard and Noe, 2015; Tsai et al., 2018; Chen et al., 2021b). According to the minimum entropy principle (Hur et al., 2019), postural fluctuations of lower entropy are less automatic due to more volitional efforts being invested in postural control (Borg and Laxaback, 2010; Roerdink et al., 2011). A low-entropy postural response is commonly seen in subjects with balance dysfunction (e.g., aged people and patients with neurological disorders) (Morrison and Newell, 2015). Hence, SV appeared to increase attentive control over the stabilometer stance in older adults. Collectively, the characteristic changes in postural fluctuations with SV were not fully compatible with the argument that greater demand is placed on visual–spatial attention with increasing motion explorations, as predicted by Hülsdünker et al. (2021). However, the behavioral observations are also not adequate to support the hypothesis of sensory reweighting for postural regulation with SV (Kim et al., 2017; Kim et al., 2020). The debates regarding SV impacts cannot be well-reconciled with the present behavioral findings.

### Stroboscopic vision-dependent modulation of EEG regional activity

In line with the postural fluctuations, SV led to wide-range alterations in local EEG in the theta, alpha, and beta bands (Figure 2A). First, SV suppressed the theta power in the mid-frontal (F_z_) and left sensorimotor areas (FC_3_, C_3_, and CP_3_) (Figure 2B, top). The mid-frontal theta oscillation, which originated from the anterior cingulate cortex, plays an important role in error elaboration and task optimization (Cavanagh et al., 2012; Pezzetta et al., 2018). When visual errors are large, mid-frontal theta power is potentiated due to unintended consequences on performance (Cavanagh et al., 2012). Previous researches showed that mid-frontal theta power is involved with the organization of goal-directed movements and postural balance maintenance (Hulsdunker et al., 2015; Malcolm et al., 2021). When a subject was guided by visual feedback during stabilometer stance, the mid-frontal theta power was enhanced to elaborate worse outcomes even though the visualized errors were faked (Chen et al., 2022). In this study, the smaller mid-frontal theta oscillation in the SV condition (Figure 2B, top) implied that fewer error contexts were perceived by the older adults with intermittent blocking of visual feedback. Correlation analysis further suggested a positive correlation between the normalized difference in theta power and the normalized difference in the RMS of the postural fluctuations (Table 2; Figure 5A). For older adults with SV, those who demonstrated greater negative ND-theta (or smaller mid-frontal theta power in the SV condition) tended to have smaller SV-induced increases in postural fluctuations (smaller postural fluctuations in the SV condition) during stabilometer stance (Figure 5A). Hence, under the condition of SV, better prediction of postural errors (or fewer unexpected visual consequences of postural performance) is a prerequisite to gaining superior stance stability, which should be achieved by compensatory upregulation of proprioceptive/vestibular inputs and a reduction of the reliance on intermittent visual feedback (Wilkins and Appelbaum, 2020; Wohl et al., 2021). Balance-related increases in theta activity could also extend to the centro-parietal regions, which are involved in sensorimotor coordination for multi-segment movements (Sipp et al., 2013). In fact, we also noted a global decrease in theta power in the left sensorimotor area with SV during stabilometer stance (Figure 2B, top), although modulation of the theta power was not significantly related to changes in postural strategy (Table 2). The decrease in the theta power of the sensorimotor area likely resulted from SV-related interference in updating of motor plans with incoming visual information, as the theta power of the area emerges during active goal-seeking behaviors (Landfield et al., 1972; Caplan et al., 2003).

Tuned to visual availability, alpha power (8–12 Hz) activity is the most prominent feature in the posterior brain (Mierau et al., 2017; Chen et al., 2021b). According to the cortical idling hypothesis, increases in alpha activity over the sensorimotor area are related to sluggish execution of movements (Pfurtscheller et al., 1996). Analogous to visual occlusion in upright stance (Goh et al., 2016), the intermittent visual feedback also brought about marked augmentation of the alpha power in the sensorimotor regions during stabilometer stance (Figure 2B, middle), in relation to less visuospatial attention for postural tracking (Chen et al., 2022). The EEG observation supported that visual reliance during stabilometer stance was downregulated by SV.

For older adults, the beta power in the fronto-centro-parietal and occipital areas was also potentiated with SV (Figure 2B, bottom). The increase in beta power with SV resembles that in the work of Peterson and Ferris (2018), who reported an increase in the occipito-parietal activity of beta power due to visual rotation during standing (Peterson and Ferris, 2018). As posterior beta oscillations were enhanced with eye closure (Tse et al., 2013), the beta potentiation with SV was likely due to the fact that the older adults sustained efforts to reweight non-visual information for stance stabilization in a situation of perceptual conflict (Lin et al., 2021). However, variations in the regional activity of the alpha and beta bands were not directly causal to the modulation of postural strategies with SV (Table 2).

### Stroboscopic vision-dependent modulation of EEG inter-regional activity

The modulation of postural strategies with SV tends to link with the enhancement of the suprathreshold connectivity in the alpha band (Tables 3, 4), which largely overlapped with the visual dorsal and frontal–occipital loops in the right hemisphere ( Figure 4, middle right). For visual search (Pantazatos et al., 2012) and the perception of complex movements (Hipp et al., 2011), the large-scale fronto-parieto-occipital connectivity is responsible for multi-sensorimotor integration of body part location and decision making (Grol et al., 2007). Especially for older adults, visual inputs are keyed to cue usage during an unstable human stance (Mierau et al., 2017; Chen et al., 2021b). There are preliminary indications that the dorsal stream is involved with the cognitive aspect of posture control under visual guidance (Takakusaki, 2017), such as analyzing movements in space and updating the motor cortex about the extra-personal space and the internal state (Raffi and Piras, 2019). Our results can be interpreted within this context. The higher alpha supra-threshold connectivity with SV could indicate stronger inhibition of the dorsal visual stream, because intermittent visual feedback was expected to interfere with the sustenance of visuospatial attention and error perception from online visual feedback. In particular, SV significantly added to stronger alpha connectivity of C_4_–Fp_2_, CP_z_–Fp_2_, and CP_4_–Fp_2_, positively linked to MF of postural fluctuations (Table 4; Figure 5B). It is known that the prefrontal cortex receives reciprocal projections from various regions, such as the premotor cortex, basal ganglia, cerebellum, and thalamus (Leh et al., 2007). Associated with enhanced activation in the supplementary motor area (SMA) and the right posterior parietal cortex, right prefrontal involvement is relevant to gaze adjustments, dynamic representation of the body schema (Pellijeff et al., 2006), and adequate allocation of visuospatial attention (Mihara et al., 2008), in preparation for a forthcoming postural perturbation. This context speaks for a positive association between SV-induced enhancement of the supra-threshold connectivity of Fp_2_ in the alpha band and increases in ND-MF (Figure 5B). The older adults with SV might down-weighed the visual channel and exhibited a high level of postural uncertainty, in that non-visual channels [such as the proprioceptive system (Lord et al., 1994)] more affected by aging could not fully compensate for the fractional impairment of the partially-blocked visual system.

One methodological concern of this study is to interpret the present data with joint effects of physical SV and SV-induced eye blinks. The number of eye blink in an experimental trial could increase in the SV condition. However, it is hard to distinguish those eye blinks exactly causal to the wearing of the stroboscopic glasses and natural eye blinks occurring roughly at a rate of 12 blinks/min (Bentivoglio et al., 1997). Despite the methodological concern, the wearing of the stroboscopic glasses substantially mediated posture sway dynamics and cortical responses during unstable stance for the elderly.

## Conclusion

Within the context of neuromechanics, the present study suggests a role for sensory reweighting with the use of stroboscopic vision for older adults during unstable stance. The changes in postural fluctuation dynamics reveals that stroboscopic vision leads to greater stance difficulty for older adults with less complex postural responses. Mid-frontal theta oscillation declines indicate fewer error contexts are perceived and corrected by the older adults due to intermittent blocking of the visual information. The selective increases in the EEG–EEG connectivity of Fp_2_ in the alpha band reflect inhibitions of visuospatial attention and functioning of the dorsal visual stream during an uneven stance. These EEG signatures explain the experimentally observed stance destabilization and postural strategy changes with greater regularity in older adults with stroboscopic vision. The corresponding cortical reorganization supports stroboscopic vision as a therapeutic approach in favor of non-visual channels for postural control of the elderly.

## Data Availability

The datasets presented in this article are not readily available because Data cannot be shared as participants were informed that their data would be stored confidentially, in accordance with the rules of the local ethics committee. Code to generate the EEG metrics is available under reasonable request. Requests to access the datasets should be directed to I-SH, ensureh@gmail.com.
